# Rhabdomyolysis in the Context of Designer Benzodiazepine Misuse

**DOI:** 10.7759/cureus.50741

**Published:** 2023-12-18

**Authors:** Greg Noe, Kaushal Shah, Taylor Quattlebaum, Sahil Munjal

**Affiliations:** 1 Department of Psychiatry, Atrium Wake Forest Baptist Health, Winston-Salem, USA

**Keywords:** drug overdose, elevated creatine kinase, substance misuse, designer benzodiazepines, acute kidney injury, rhabdomyolysis, designer drugs

## Abstract

Designer benzodiazepines belong to a class of lab-created psychoactive compounds, with limited federal regulation, no toxicity testing, and reported high potency, leading to substantial overdose risk and harmful clinical syndromes. Benzodiazepine misuse has been previously documented to be associated with rhabdomyolysis, with elevated creatine kinase (CK) during and after acute episodes of intoxication. Here, we present a case of profound rhabdomyolysis and associated acute kidney injury (AKI) after acute designer benzodiazepine intoxication.

A 26-year-old male with a history of poly-substance misuse, including alcohol, psychedelics, opiates, kratom, and benzodiazepines, presented to the emergency department with altered mental status and agitation after an accidental overdose on liquid flubromazolam and clonazolam, designer benzodiazepines purchased online. He went on to develop seizure-like activity. Additional labs revealed AKI with creatinine 2.22 mg/dL (reference 0.74-1.35 mg/dL, baseline 0.88 mg/dL). He was discovered to have severe rhabdomyolysis that peaked at 131,920 U/L (reference 55-170 U/L) on the fourth day of admission.

This case demonstrates the potential deleterious effects of the designer benzodiazepine class, including prolonged sedation, AKI, and severe rhabdomyolysis. In addition, seizure-like manifestations may occur during the intoxication or withdrawal phase. Designer benzodiazepines may produce rhabdomyolysis; however, the mechanism is unknown. Direct myotoxicity or prolonged immobilization may be contributors to rhabdomyolysis. More research is needed to elucidate the consequences of designer benzodiazepine misuse. Clinicians should be aware of their use given the ease of availability online and rising popularity.

## Introduction

Designer benzodiazepines, such as clonazolam and flubromazolam, are a class of lab-created triazolobenzodiazepines, both of which have been temporarily designated as Schedule 1 controlled substances by the Drug Enforcement Administration (DEA) as of July 6, 2023. These compounds have no safety or toxicity testing and unknown potency, leading to unpredictable overdose risk and novel clinical manifestations [1,2]. These synthesized benzodiazepine analogs belong to a larger class of novel psychoactive substances (NPS, or “designer drugs”) that are primarily being produced in clandestine laboratories, and designed to circumvent drug regulatory legislation [3]. To date, there are a limited number of case reports documenting acute designer benzodiazepine overdose. There exists one case report involving diclazepam and one case report of a flubromazolam overdose, both documenting profound intoxication and adverse effects, including rhabdomyolysis and deep coma with mild hypoxic brain injury [1,4]. Benzodiazepine misuse has been previously associated with the clinical syndrome of rhabdomyolysis, with elevated creatine kinase (CK) during and after acute episodes of intoxication [5]. Additionally, benzodiazepine use has a risk of associated addiction, tolerance, and withdrawal effects, including anxiety, depression, tremor, insomnia, seizures, and altered mental status [6]. Here, we present a case of severe rhabdomyolysis and AKI associated with acute designer benzodiazepine intoxication.

## Case presentation

A 26-year-old male with a history of poly-substance misuse, including alcohol, psychedelics, opiates, kratom, and benzodiazepines, presented to the emergency department with altered mental status and agitation after an accidental overdose of flubromazolam and clonazolam. He initially had a temperature of 39 °C, tachypnea (respiratory rate 22/min), and otherwise stable vital signs (blood pressure 126/78 mmHg, pulse 64 beats per minute, oxygen saturation 100%), and received antipyretic treatment with acetaminophen 650 mg rectal suppository. Upon arrival, the patient was making incoherent noises and was not verbally responding to questions. He was able to move his arms and legs in an uncoordinated, non-purposeful manner. Cranial nerves were grossly intact, and he was intermittently opening his eyes. The patient was markedly altered and unable to protect his airway. Therefore, he was intubated with the use of etomidate and rocuronium, and the endotracheal tube was secured with an inflatable cuff to decrease the chance of aspiration. Upon further evaluation, he developed seizure-like activity, including tonic-clonic muscle activity of the upper and lower extremities, without apnea, involuntary urinary or fecal voiding, sialorrhea, or tongue laceration. He required several serial doses of midazolam (2 mg, 2 mg, 4 mg, 4 mg, 4 mg, and 4 mg) totaling 20 mg, which resolved the tonic-clonic activity. Due to ongoing concerns for CNS depressant withdrawal and agitation, he was deeply sedated with a propofol drip. No EEG activity was recorded during this episode to confirm seizure activity.

Initial screening labs were obtained. A urine drug screen (UDS) was negative for benzodiazepines and alcohol and positive for tetrahydrocannabinol (THC) and buprenorphine, a home medication. Acetaminophen, ethanol, and salicylate levels were negative. Initial glucose was normal at 97 mg/dL. Additional labs revealed leukocytosis of 22,500 white blood cells per milliliter, AKI with creatinine 2.22 mg/dL, an elevated anion gap to 20 mEq/L (normal <12-14 mEq/L), and a lactate of 2.52 mmol/L (normal <2 mmol/L). His blood urea nitrogen (BUN) to creatinine ratio was 9.46 (BUN 21 mg/dL). Urinalysis was positive for 3+ blood, negative for nitrites, and negative for leukocytes. He developed severe rhabdomyolysis, with an initial CK of 3,910 U/L that peaked at 131,920 U/L on day four of admission. Acute elevation of alanine transaminase (ALT) and aspartate transaminase (AST) co-occurred with a peak ALT of 326 and AST of 1033 on day 4.

The initial history was obtained from the patient’s mother (a nurse) who endorsed he had been using liquid flubromazolam and clonazolam in unknown quantities, which he purchased online. Possible co-ingestions included psilocybin mushrooms and kratom, which were also found in his personal belongings. It is unknown if the patient also ingested these substances in the 24 hours prior to presentation. He was difficult to rouse home over the prior 24 hours, with intermittent periods of her hearing him become agitated, awakening, and yelling, followed by a resumption of minimal responsiveness. He subsequently rolled off the bed, sustaining a left forehead contusion, at which time his mother called emergency medical services. To rule out intracranial pathology, a CT head and cervical spine was performed and was negative for acute pathology, with normal lumbar puncture.

Due to concern for possible meningitis, the patient was initiated on broad-spectrum antibiotics vancomycin 1750 mg in 287.5 mL of normal saline and ceftriaxone 1 g in 100 mL of normal saline. Acyclovir 750 mg in 250 mL normal saline was administered for empiric coverage of viral meningitis or encephalitis. Propofol was used for continuous sedation in the first 24h period after the emergency department evaluation. After weaning propofol, he was awake, alert, and conversational without any focal or non-focal neurologic deficit on evaluation 36 hours after the initial presentation. On hospital day 2, he had a period of rebound sedation. During this period, he was difficult to rouse, with hyper-somnolence, and not consistently responding to questions or commands. Vital signs were within normal limits at this time. For AKI secondary to rhabdomyolysis, the patient was given several liters of normal saline and lactated Ringer’s solution. The patient’s mental status gradually returned to baseline on day 5. He was coherent, conversational, answering questions appropriately, and exhibited no confusion, delirium, or sedation. He reported no feelings of intoxication. Collateral history from the patient’s mother confirmed that the baseline was at his neurologic baseline. No neurologic deficits were observed. He was discharged on day 9 with a CK of 4,494 U/L and referred to outpatient medication-assisted treatment.

## Discussion

Benzodiazepine misuse is commonly encountered by clinicians, representing the second most common drug category of overdose in adult patients in the United States [7]. Designer benzodiazepines, lab-created chemical derivatives of medically prescribed benzodiazepines, are less frequently encountered and not approved for medical use. The first designer benzodiazepines appeared around 2007, with up to 29 compounds ultimately being created, some with very high potency [8]. In 2012, designer benzodiazepines became more widely available via multiple online vendors [8]. Flubromazolam and clonazolam are both highly potent designer benzodiazepines in the triazolo-benzodiazepine class, similar to alprazolam and triazolam [1,2,9,10]. Flubromazolam has resulted in a standard UDS positive for benzodiazepines, but many designer benzodiazepines may not show up on standard UDS [1,2,10]. Since diclazepam metabolizes to lorazepam, it is detectable on a standard UDS as seen in the Runnstrom et al. case report [4].

While this patient had an extreme elevation in creatine kinase (131,920 U/L peak), he had only a modest elevation in creatinine (2.22 mg/dL), with a normal BUN to creatinine ratio (9.46, normal ratio <10:1). The patient had 3+ blood/hemoglobin in his urinalysis with an absence of red blood cells. This renal injury pattern suggests intrinsic renal injury secondary to rhabdomyolysis [11]. Rhabdomyolysis releases myoglobin, which filters through the nephron and combines with Tamm-Horsfall (THP) to form obstructive tubular casts, a form of intrinsic renal injury via acute tubular necrosis [11]. Additionally, reactive oxygen species formed from hydrogen peroxide and an abundance of free ferrous iron contribute to intrinsic renal injury [11]. It is unclear if there is a significant pre-renal component to this patient’s AKI, but a normal BUN to creatinine ratio would suggest that there is likely no significant contribution from volume depletion. The patient’s transaminase pattern of a peak ALT of 326, and AST of 1033 on day 4 (approximate 3:1 AST/ALT ratio) suggest acute liver injury from drug toxicity with superimposed rhabdomyolysis, as AST is found in high concentrations in skeletal muscle [12].

There is a paucity of data detailing designer benzodiazepine’s potency, toxicity, and other related deleterious clinical effects. To date, there has been only one published case of documented rhabdomyolysis involving an overdose of a designer benzodiazepine, flubromazolam, with elevated serum CK at 15, 960 U/L (2). Limited literature exists on rhabdomyolysis associated with designer benzodiazepines as a class, including clonazolam. Rhabdomyolysis may be due to direct myotoxicity, prolonged immobilization, or a combination of these two mechanisms. Regardless of the cause, early detection and treatment of rhabdomyolysis are critical to limiting renal damage and other sequelae. Treatment of rhabdomyolysis involves early aggressive intravenous (IV) fluids, with a urine output goal of 300 mL/h, and correcting electrolyte derangements [13].

Limitations of this case report include the absence of therapeutic drug monitoring, mass spectroscopy, or gas chromatography confirming designer benzodiazepine presence and quantity in the patient's blood. Quantitative liquid chromatography has been shown to be able to quantify flubromazolam in both serum and urine but was not performed in this case [2]. The patient was unable to recall the dosages and timing of dosages of flubromazolam and clonazolam. Due to the degree of intoxication of the patient over the days prior to presentation, he lacks a clear memory of what other substances he may have taken over this period. He did endorse the use of his prescribed buprenorphine for opiate use disorder, which was positive in his urine drug screen. Beyond a standard UDS, additional toxicologic analysis to detect other co-ingestants was not performed, so there is the possibility of an unknown contribution to rhabdomyolysis from another substance. Additionally, buprenorphine may also have CNS depressant properties and possibly contributed to this patient's altered mental status and sedation, confounding the clinical picture. The pharmacokinetics and pharmacodynamic properties of designer benzodiazepines are largely unknown, but user reports suggest high potency (Table 1) [8]. Flubromazolam has demonstrated a possible tri-phasic serum concentration pattern in a single researcher-volunteer, who ingested 0.5 mg, explaining the possibility of rebound sedation [9]. Flubromazolam’s serum peak concentrations were recorded at five hours post-ingestion at 7.4 ng/mL, eight hours post-ingestion at 8.6 ng/mL, and 30 hours post-ingestion at 5.2 ng/mL [9]. Half-life was estimated at 10-20 hours [9]. Case reports illustrate the dangerous clinical syndromes that can result from overdose, including rhabdomylosis and AKI, hypoxic brain injury, and potential seizure-like sequelae.

**Table 1:** Typical dose, onset, duration, after-effects, and commonly reported experiences associated with flubromazolam and clonazolam [1,2,9,10]

	Typical Dose	Onset	Duration	After Effect	Commonly reported experiences
Flubromazolam	100 ug-600 ug	20-45 min	6-12 hr	6-12 hr; Rebound sedation >24 hours	Significant sedation and anxiolytic effects. Reports of partial amnesia. Case report of coma and respiratory failure.
Clonazolam	75 ug-1000 ug	10-30 min	6-10 hr	1-12 hr	Sedation, anxiolytic effect, amnesia, lethargy/drowsiness, slurred speech, tachycardia. Difficult to dose and potent at low doses.

## Conclusions

This case demonstrates the potential dangers of designer benzodiazepine abuse, including prolonged sedation, CNS depressant withdrawal syndrome, possible seizures, and severe rhabdomyolysis with AKI. These compounds have the potential to induce profound CNS depression and cause life-threatening adverse effects. Research is limited to a small number of case reports, and more studies are needed to explore the consequences of designer benzodiazepine use further. Clinicians should be aware of their use and clinical presentation, given the ease of availability and increasing frequency of use.
